# The Baader-Meinhof Phenomenon of Dieulafoy’s Lesion

**DOI:** 10.7759/cureus.4595

**Published:** 2019-05-03

**Authors:** Sindhura Kolli, Khoi Paul Dang-Ho, Amit Mori, Krishna Gurram

**Affiliations:** 1 Internal Medicine, The Brooklyn Hospital Center, Affiliate of the Mount Sinai Hospital, Brooklyn, USA; 2 Gastroenterology, The Brooklyn Hospital Center, Affiliate of the Mount Sinai Hospital, Brooklyn, USA

**Keywords:** occult gi bleeding, dieulafoy's lesion, dieulafoy

## Abstract

Despite modern investigative innovations in the cutting edge field of gastroenterology, we are reminded of our contemporary limitations when we encounter the ever evasive Dieulafoy’s lesion (DL). Ever since it has been initially described in 1884, its rare but frustrating presence creates a calamitous situation. Even more so when it presents atypically, much like it did in our patient. This review of DL delves into the history, epidemiology, characteristics, the most current and innovative diagnostic measures available, as well as treatment and prevention of recurrence of these obscure gastrointestinal (GI) bleeding sources.

## Introduction and background

There is a cognitive bias when one has a new experience or learned something new and suddenly one begins to encounter it more frequently, even if its been there all along. This phenomenon, known as Baader-Meinhof, becomes most crucial in the dissemination of academic literature about rare entities such as Dieulafoy’s lesion (DL). By parlaying information about DLs to investigators or endoscopists, it forces them to not only intelligibly discern these esoteric submucosal defects that can readily go unnoticed, but also actively search for it with every withdrawal of the scope to determine the etiology behind obscure gastrointestinal (GI) bleeding. This review will serve to explain the history, epidemiology, characteristics, the most current and innovative diagnostic measures available, as well as treatment and prevention of recurrence of DLs.

**Figure 1:**
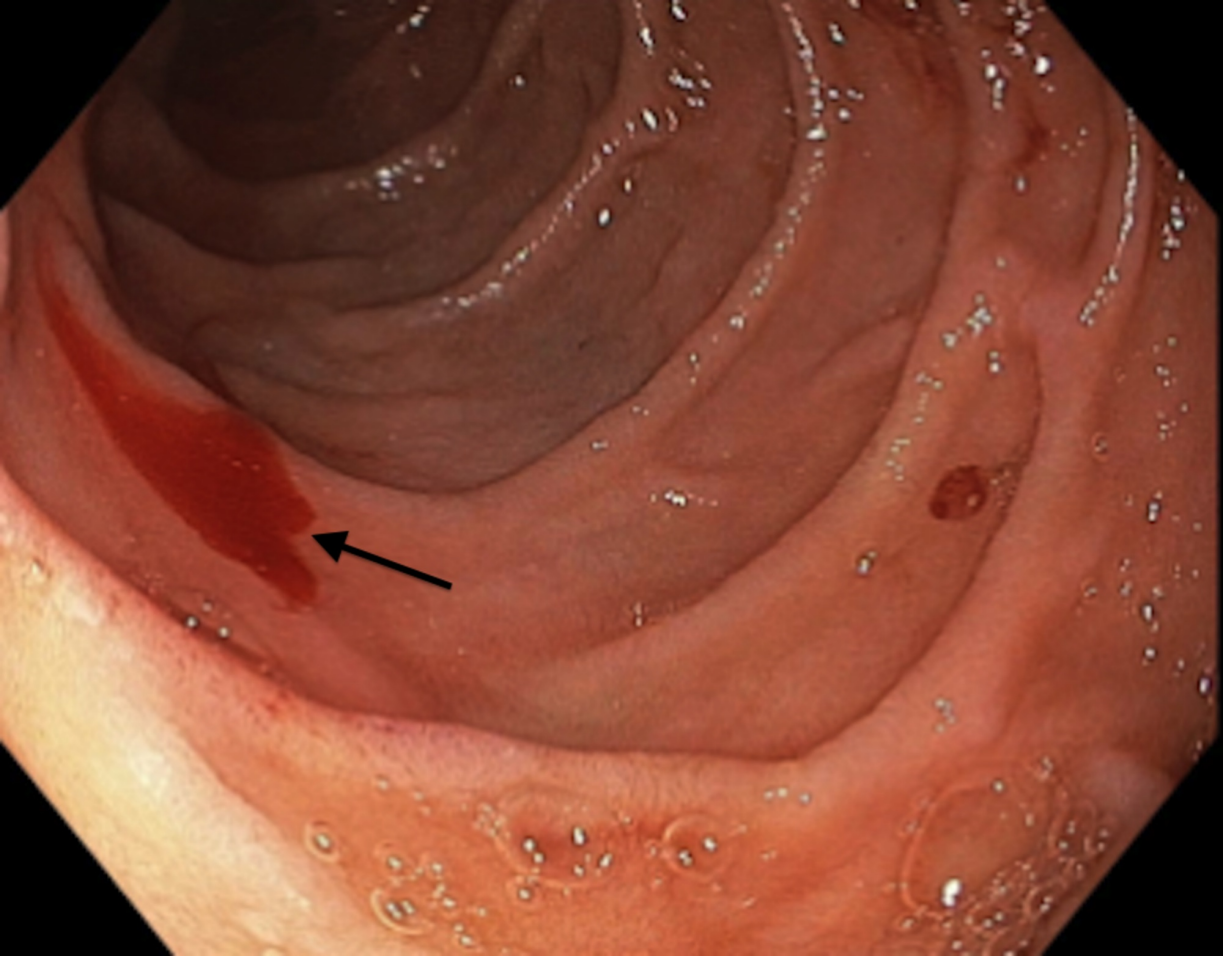
Bleeding periampullary Dieulafoy's lesion

**Figure 2:**
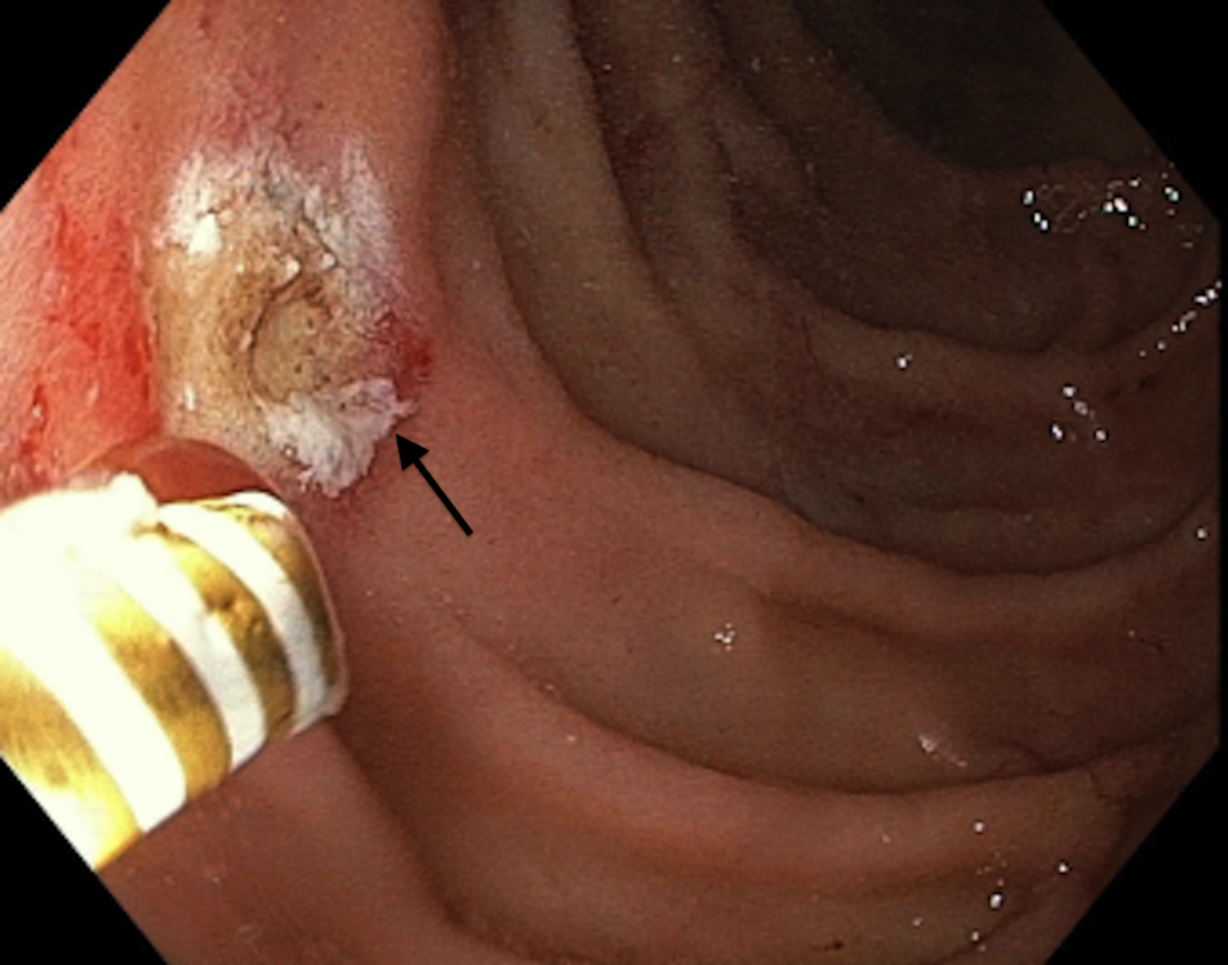
Primary hemostasis of Dieulafoy's lesion through electrocoagulation

An 87-year-old female patient with a history of gastroesophageal reflux disease (GERD), gastritis, diverticulosis and chronic non-steroidal anti-inflammatory drugs (NSAID) use presented with melena, diffuse abdominal pain and non-bloody, non-bilious emesis. In the setting of her acute anemia with hemoglobin (Hb) of 5.9 g/dL and tachycardia, an esophagoduodenoscopy (EGD) demonstrated a single medium-sized DL in the second portion of the duodenum next to the major papilla oozing blood that was controlled with a bipolar diathermy (Figures 1-2). Daily proton pump inhibitors (PPIs), discontinuation of NSAIDs, and appropriate outpatient follow-up were recommended.

## Review

DLs are dilated submucosal veins that are exposed to the surface and endoscopically visualized as bleeding points without surrounding erosions or ulcerations. It accounts for 1% to 5.8% of cases of acute nonvariceal upper GI bleeding [1-2] (Poster: Kolli S, Mori A, Gurram K. A Rare Dieulafoy Lesion in the Periampullary Duodenum. American College of Gastroenterology Conference; Oct 2018). Despite being initially described by Dr. M.T. Gallard in 1884, the nomenclature for this lesion is attributed to Georges Dieulafoy in 1898 for detailing seven patients with this pathology. He theorized that they were the initial stages of a common gastric ulcer interrupted by erosive bleeding and are now widely known as DLs [3-5].

Histologically, an alternative nomenclature for DL is a “caliber-persistent artery,” due to the submucosal artery failing to narrow to rival the normal capillary microvasculature of the mucosa otherwise known as ramification. Instead, it maintains a diameter 10 times the size compared to normal mucosal arteries, leading to profuse bleeding when irritated or under pressure during normal peristalsis [4]. 

These lesions can affect any age group but present more commonly in the older population, ranging from the fifth to the seventh decade of life [1,6-8]. The lesion is more prominent in males than females with a ratio of 2:1 [1,6-7]. Comorbidities are present in up to 90% of patients [7]. The most common include cardiovascular disease, hypertension, diabetes mellitus, liver disease, and renal failure [6-7]. Patients with DL might also present with a history of NSAIDs or anticoagulant use which can promote bleeding [6]. No causal link, however, has been established between DLs and the use of NSAIDs, alcohol use, tobacco use, presence of peptic ulcer disease or Helicobacter pylori (H.pylori) infection [4,6,9].

Around 70% of DLs are located in the stomach. Within the gastric region, most of the lesions (75%) can be found in the proximal stomach, particularly 6 cms from the gastroesophageal junction [1,6]. Lesions can also occur further along the GI tract including the duodenum, jejunum, ileum, cecum, appendix, colon, and anal canal which can present as lower GI bleeding. DLs can also present in extra-gastrointestinal regions such as the bronchus, leading to hemoptysis [6-7]. A high proportion of DLs were found at intestinal anastomoses, predominantly Billroth II anastomosis after gastrectomy [1,4].

Patients with DLs are typically asymptomatic but then can present acutely with massive GI hemorrhage [1,6-8]. Bleeding is typically reported as melena (44%), hematemesis (30%), a combination of both (18%), hematochezia (6%), or as iron deficiency anemia (1%) [1,5-6]. Patients can present with signs of hemodynamic instability such as tachycardia, hypotension, and orthostasis, along with acute prerenal azotemia [6]. Hemoglobin levels typically range between 8.4 to 9.2 g/dL [6-7]. Recurrent bleeding < 72 hours after initial presentation can occur if left untreated following the initial endoscopy, encouraging prompt diagnosis [6]. Other GI symptoms, especially abdominal pain, are uncommon in patients with DL, and if present, usually indicates an alternate diagnosis such as peptic ulcer disease or complications from bleeding such as mesenteric ischemia secondary to hemorrhagic shock [6].

Diagnosis remains challenging as urgent EGD is indicated, but without an ulcer base to guide identification, diagnosis on an initial endoscopy can be as low as 49%; 33% of those affected require a repeat endoscopy and 18% require an exploratory laparotomy [9]. False negatives can be attributed to a small bleeding point, intermittent bouts of bleeding, lack of anatomical identifiers, or excessive bleeding concealing the lesion [4,9]. Proper diagnosis can involve multiple endoscopies, the use of push enteroscopy which is an extension of an EGD, side-viewer endoscope, or an exploratory laparotomy which provide diagnostic and therapeutic utility. Diagnostic modalities without the advantage of immediate intervention include wireless capsule endoscopy, angiography, or technetium-99m labelled bleeding scans when searching in anatomically inaccessible locations [1,4,6,9] (Poster: Kolli S, Oct 2018). Agreed upon criteria for accurate diagnosis of a DL include the following: 1) active arterial spurting or micro-pulsatile streaming of blood from a minute (<3 ram) mucosal defect or through normal surrounding mucosa; 2) visualization of a protruding vessel, with or without active bleeding, within a minute mucosal defect or through normal surrounding mucosa; or 3) fresh, densely adherent clot with a narrow point of attachment to a minute mucosal defect or to normal-appearing mucosa [1,4,6,10].

Treatment is a two-pronged approach since the recurrence rate of a DL can range from 9% to as high as 40%. Primary hemostasis can be achieved with electrocoagulation, which provides a quick and inexpensive solution with an almost 80% hemostatic success rate [11]. Equivocal methods also include sclerotherapy with ethanol or norepinephrine, thermocoagulation, argon plasma coagulation or hemostatic clips [1,4,6,11] (Poster: Kolli S, Oct 2018). Secondary hemostasis can be accomplished through repeat endoscopy, angiography, or rarely, surgical wedge resection. Tattooing the site of original therapeutic intervention during the initial endoscopy steers the secondary hemostatic measures [11]. With proper diagnosis and treatment, the rate of mortality has decreased from 30% in the 1970s to currently 8%; however, the difficulty lies in vigilant detection, especially in anomalous locations, as was the case in our patient [6] (Poster: Kolli S, Oct 2018).

## Conclusions

DLs incur healthcare costs from numerous tests and hospital visits and increases aggravation due to its difficulty in diagnosis and delay in prompt treatment. Numerous available modalities, such as push enteroscopy as an extension of an EGD and side-viewer endoscope, aid in diagnosing DLs in anatomically difficult locations. However, the ability to recognize them lies in the endoscopist's scope of discernation. We hope this review coupled with the Baader-Meinhof phenomenon increases the perception of this rarely occurring pathology.
